# Complete Mitochondrial DNA Sequence of the Mucoralean Fungus *Absidia glauca*, a Model for Studying Host-Parasite Interactions

**DOI:** 10.1128/genomeA.00153-16

**Published:** 2016-03-24

**Authors:** Sabrina Ellenberger, Anke Burmester, Johannes Wöstemeyer

**Affiliations:** General Microbiology and Microbe Genetics, Friedrich-Schiller-University Jena, Jena, Germany

## Abstract

The mitochondrial DNA (mtDNA) of *Absidia glauca* has been completely sequenced. It is 63,080 bp long, has a G+C content of 28%, and contains the standard fungal gene set. *A. glauca* is the recipient in a laboratory model for horizontal gene transfer with *Parasitella parasitica* as a donor of nuclei and mitochondria.

## GENOME ANNOUNCEMENT

*Absidia glauca* is a well-studied genetic model among mucoralean fungi. Especially appealing is the interaction with the mycoparasite *Parasitella parasitica*. This biotrophic parasitism involves the fusion of specialized hyphal tips with the host *A. glauca* and other *Mucor* relatives (1). The transfer and expression of nuclear genes have been described for several genes involved in amino acid biosynthesis or that confer antibiotic resistance (2, 3). After infection, auxotrophic mutants of *A. glauca* are complemented by their prototrophic counterparts of the parasite. In order to initiate the analysis of mitochondrial information after transfer, we sequenced the mitochondrial DNA (mtDNA) of both *A. glauca* and *P. parasitica* (4).

The *Absidia* chondriome was revealed by Illumina sequencing (Eurofins Genomics, Ebersberg, Germany). The mitochondrial information resides on a single circular molecule with a total length of 63,080 bp. Compared with others, *A. glauca* is among fungi with the largest mtDNA. For other zygomycetes, lengths differ by 54,178 bp in *Rhizopus oryzae* (5) and 62,082 bp in *Phycomyces blakesleeanus* (accession no. KR809878).

The *Absidia* chondriome harbors genes for those proteins that are normally found in fungi, the small and large subunit rRNAs, and 24 tRNAs. An unusual feature of the *A. glauca* chondriome is the cluster of tRNAs, which encompasses all tRNA genes. On the whole, 29 protein-coding genes were identified, 12 of which are split or duplicated (*atp9*, *cob, cox1*, *cox2*, *cox3*, *nad1*, *nad3*, *nad4*, *nad5*, *nad6*, *nad2*, and *trnM*). Both DNA strands are transcribed.

The chondriome harbors 12 genes for endonucleases (four with the LAGLIDADG domain and eight with the GYI-YIG domain). Most of them are intron situated (9 of 12) and thus must be addressed as true homing endonucleases. The number of endonucleases in other zygomycetes ranges between zero in the opportunistic human pathogen *Lichtheimia ramosa* (6) and 12 in *Phycomyces blakesleeanus*. Remarkably, the chondriome of the host *P. parasitica* contains 27 endonucleases.

### Nucleotide sequence accession number.

The mtDNA sequence is deposited in GenBank under accession no. KU196782.

## References

[1] Burgeff H 1924 Untersuchungen über Sexualität und Parasitismus bei Mucorineen. Bot Abhandl 4:5–135.

[2] Kellner M, Burmester A, Wöstemeyer A, Wöstemeyer J 1993 Transfer of genetic information from the mycoparasite *Parasitella parasitica* to its host *Absidia glauca*. Curr Genet 23:334–337. doi:10.1007/BF00310895. 8467531

[3] Burmester A, Karimi S, Wetzel J, Wöstemeyer J 2013 Complementation of a stable *met-2–1* mutant of the zygomycete absidia *glauca* by the corresponding wild-type allele of the mycoparasite *Parasitella parasitica*, transferred during infection. Microbiology 159:1639–1648. doi:10.1099/mic.0.066910-0. 23704789

[4] Ellenberger S, Burmester A, Wöstemeyer J 2014 Complete mitochondrial DNA sequence of the mucoralean fusion parasite *Parasitella parasitica*. Genome Announc 2(6):e00912-14. doi:10.1128/genomeA.00912-14. 25395626 PMC4241652

[5] Seif E, Leigh J, Liu Y, Roewer I, Forget L, Lang BF 2005 Comparative mitochondrial genomics in zygomycetes: bacteria-like RNase P RNAs, mobile elements and a close source of the group I intron invasion in angiosperms. Nucleic Acids Res 33:734–744. doi:10.1093/nar/gki199. 15689432 PMC548346

[6] Leung S-Y, Huang Y, Lau SKP, Woo PCY 2014 Complete mitochondrial genome sequence of *Lichtheimia ramosa* (syn. *Lichtheimia hongkongensis*). Genome Announc 2(4):e00644-14. 24994796 10.1128/genomeA.00644-14 PMC4081996

